# Adenosine Triphosphate Stimulates *Aquifex aeolicus* MutL Endonuclease Activity

**DOI:** 10.1371/journal.pone.0007175

**Published:** 2009-09-24

**Authors:** Jerome Mauris, Thomas C. Evans

**Affiliations:** New England Biolabs, Inc., Ipswich, Massachusetts, United States of America; University of Massachusetts Medical School, United States of America

## Abstract

**Background:**

Human PMS2 (hPMS2) homologues act to nick 5′ and 3′ to misincorporated nucleotides during mismatch repair in organisms that lack MutH. Mn^++^ was previously found to stimulate the endonuclease activity of these homologues. ATP was required for the nicking activity of hPMS2 and yPMS1, but was reported to inhibit bacterial MutL proteins from *Thermus thermophilus* and *Aquifex aeolicus* that displayed homology to hPMS2. Mutational analysis has identified the DQHA(X)_2_E(X)_4_E motif present in the C-terminus of PMS2 homologues as important for endonuclease activity.

**Methodologies/Principal Findings:**

We examined the effect ATP had on the Mn^++^ induced nicking of supercoiled pBR322 by full-length and mutant *A. aeolicus* MutL (Aae MutL) proteins. Assays were single time point, enzyme titration experiments or reaction time courses. The maximum velocity for MutL nicking was determined to be 1.6±0.08×10^−5^ s^−1^ and 4.2±0.3×10^−5^ s^−1^ in the absence and presence of ATP, respectively. AMPPNP stimulated the nicking activity to a similar extent as ATP. A truncated Aae MutL protein composed of only the C-terminal 123 amino acid residues was found to nick supercoiled DNA. Furthermore, mutations in the conserved C-terminal DQHA(X)_2_E(X)_4_E and CPHGRP motifs were shown to abolish Aae MutL endonuclease activity.

**Conclusions:**

ATP stimulated the Mn^++^ induced endonuclease activity of Aae MutL. Experiments utilizing AMPPNP implied that the stimulation did not require ATP hydrolysis. A mutation in the DQHA(X)_2_E(X)_4_E motif of Aae MutL further supported the role of this region in endonclease activity. For the first time, to our knowledge, we demonstrate that changing the histidine residue in the conserved CPHGRP motif abolishes endonucleolytic activity of a hPMS2 homologue. Finally, the C-terminal 123 amino acid residues of Aae MutL were sufficient to display Mn^++^ induced nicking activity.

## Introduction

Despite their incredible accuracy, replicative polymerases are known to make misincorporation, deletion, and addition errors [1]. In addition to a 3′-5′ exonuclease activity to improve replication fidelity, there is a pathway termed the mismatch repair pathway (MMR) present in both prokaryotes and eukaryotes to correct DNA mismatches and small insertions/deletions (ID) before they lead to permanent mutation [2], [3]. The general mechanism of mismatch repair involves the steps of mismatch recognition, DNA nicking 5′ and 3′ to the mismatch, mismatch strand removal, and DNA synthesis to correct the mistake. These steps are well conserved from bacteria to multicellular eukaryotes. There is, however, an interesting divergence in how the newly synthesized DNA strand that contains the misincorporated base is discriminated from the mother strand.

The first mechanism of daughter/mother strand discrimination is used by the well-studied bacteria *Escherichia coli* and takes advantage of the transient hemimethylation state of its DNA during DNA replication. Following nucleotide misincorporation during DNA replication, MutS, the “sensor” molecule of the mismatch repair pathway, binds to the DNA mismatch [4], [5]. This triggers ATP/ADP exchange in MutS that then stimulates binding of MutL [6]. MutL also binds ATP [7], [8] and acts as a bridge between MutS and the endonuclease MutH [9]. MutH recognizes dam methylation sites and, when stimulated by the mismatched DNA/MutS/MutL complex, nicks hemimethylated dam sites on the newly synthesized, unmethylated strand [10]. The nicks direct subsequent repair steps.

The second mechanism is best understood from studies of eukaryotic mismatch repair, but it also appears to occur in bacteria that lack MutH homologues. In humans there are MutS homologues that serve as mismatch sensors in common with the *E. coli* pathway [11]. MutL homologues are found in humans; however, no homologues of MutH have been found. Recent studies have demonstrated that homologues of the PMS2 subunit of human MutLα nick the daughter strand 5′ and 3′ to a mismatch thus taking on the role that MutH plays in *E. coli* [12], [13], [14]. Interestingly, strand discrimination does not appear to occur by methylation status, but instead by the presence of a preexisting nick near the mismatch. The nick-directed endonuclease activity of hPMS2 and related proteins, and therefore repair, relies on discontinuities being more prevalent in the newly synthesized DNA strand.

The most highly studied MutL protein is the one found in *E. coli*. It has been reported to exist as a homodimer in solution with each monomeric subunit composed of 3 domains. The N-terminal domain (LN40) carries a ATPase domain which belongs to the Gyrase Hsp Histidine Kinase MutL (GHKL) ATPase/Kinase superfamily [7], [15]. The binding of ATP triggers a dramatic conformational change in this polypeptide that promotes the self-dimerization of the LN40 domains [7], [16]. This domain has many roles including involvement in MutL protein and DNA binding activity and activation of the endonuclease MutH. The self-dimerization of MutL LN40 domains also increases the affinity of MutL for DNA by creating a positively charged cleft [16]. The second domain is an 8 kD-linker that bridges LN40 with the 20 kD C-terminal domain (LC20). LC20 is involved in MutL homodimerization even in the absence of ATP [17], [18].

Eukaryotic MutL proteins, such as human MutLα (hMutLα) are heterodimeric proteins [3]. Human MutLα is composed of MLH1 and PMS2 subunits. The N-terminal domain of hPMS2 is homologous to the LN40 region in *E. coli* MutL, however, the C-terminal domains vary significantly between these two proteins. It is in the C-terminal domain of the hPMS2 subunit that a conserved DQHA(X)_2_E(X)_4_E heavy metal binding site was discovered [13], [19], [20]. Mutations in this motif were found to abolish endonuclease activity in hMutLα and *Saccharomyces cerevisiae* MutLα [12], [13]. A second conserved motif, the CPHGRP motif, has been identified in the C-terminal domain of hPMS2 homologues. Mutation of the first Cys in this motif in *Thermus thermophilus* MutL (Tth MutL) abolished sensitivity to ATP but not its endonuclease activity [21]. Furthermore, changing the conserved His or Arg residues inhibited MMR in an *in vitro* system [20]

MutL in both the methyl-directed and nick-directed MMR pathways is regulated by the binding of ATP. It has been shown in *E. coli* that the binding of ATP dramatically changes the conformation of MutL and induces the dimerization of the N-terminal LN40 domain changing the MutL from a “loose” form to a “tight” one [7]. The binding of ATP or a non-hydrolyzable ATP analogue induces the physical interaction between MutL and the endonuclease MutH. A truncated MutL that lacks the LC20 domain also shows the same property. The binding of ATP by *E. coli* MutL correlates with activation of MutH. Interestingly, while the Mn^++^ induced nicking activity of hMutLα and yMutLα are significantly stimulated by ATP [12], [13], it has been reported that the homologous proteins in *T. thermophilus* and *A. aeolicus* are down regulated by ATP [21]. Perhaps hPMS2 homologues are regulated differently in eukaryotes and prokaryotes.

The aim of this study is to better understand the ATP regulation of bacterial hPMS2 homologues and gain a greater understanding of the roles that the DQHA(X)_2_E(X)_4_E and CPHGRP motifs play in MutL function. Initial rate studies of the Mn^++^ induced nicking activity of Aae MutL demonstrated that ATP has a stimulatory effect. A truncation protein composed of only the C-terminal 123 amino acid residues of Aae MutL (Aae MutL-CTD) retained endonucleolytic activity. We also demonstrate for the first time, as far as we are aware, that the conserved histidine residue in the CPHGRP motif is required for MutL activity. This data implies that Aae MutL responds to ATP in a manner similar to hPMS2 and the information garnered herein may be applicable to all hPMS2 homologues.

## Materials and Methods

### Cloning wild-type and mutant Aae MutL

The gene encoding *A. aeolicus* MutL (accession number AAC07483) was amplified by the Touchdown method [22] of the polymerase chain reaction from *A. aeolicus* genomic DNA using Phusion™ DNA polymerase (New England Biolabs, Inc., Ipswich, MA) and the forward and reverse primers 5′-GCGGCGGCGGC GGCCTCGAGCATATGTTTGTAAAGTTACTTCCTCC-3′ and 5′-CGGCGGCGGATCCGCTCTTCCGC AGTAATTCCTGCCTACTTTTTCG-3′, respectively. The *A. aeolicus* genomic DNA was the kind gift of Dr. Isaac Cann at the University of Illinois. The forward and reverse primers incorporated NdeI and SapI restriction sites, respectively, for subcloning into the pTWIN1 vector [23] (New England Biolabs, Inc.). The PCR product was digested with NdeI and SapI restriction endonucleases (New England Biolabs, Inc) and subcloned into the same sites in pTWIN1 to create a direct fusion with the Mxe GyrA Intein-tag.

Aae MutL-CTD and Aae MutL mutants E357K, H404A and R406A were generated using the Phusion™ Site-Directed Mutagenesis Kit (New England Biolabs, Inc.). Aae MutL-CTD utilized primers 5′-pAAATTCAAGGAGGTGCTGGTCCACGAAGTACA-3′ and 5′-pAGG ATAAACTACGAAAAACTGAAGGACGAAAACTTAGCCTGC-3′, Aae MutL (E357K) used primers 5′-pAAATTCAAGGAGGTGCTGGTCCACGAAGTACA-3′ and 5′-pAGGATAAACTACGAAAAACTGAAGG ACGAAAACTTAGCCTGC-3′, Aae MutL (H404A) used primers 5′-pGGAAGACCCATATACTACAAAATACCCCT-3′ and 5′-pCGCGGGGCAAACGTGGGATTTTCC-3′, and Aae MutL (R406A) utilized primers 5′-pCCCATATACTACAAAATACCCCTGAGGG-3′ and 5′-pCGCTCCGTGGGGGCAAACG TGGG-3′. The resulting mutant Aae MutL genes were present as direct fusions with the gene encoding the Mxe GyrA Intein-tag. Wild-type *E. coli* MutL was cloned as a direct fusion with the Sce VMA1 Intein-tag in the pTYB1 plasmid [24] (New England Biolabs, Inc). This expression plasmid was the generous gift of Dr. Huimin Kong, BioHelix, Corp.

### Protein purification

The appropriate MutL vectors were used to transform chemically competent *E. coli Rosetta*™ (DE3) strains (Novagen ®, Madison, WI) (*F^−^ ompT hsdS_B_(r_B_^−^ m_B_^−^) gal dcm (DE3) pRARE2 (Cam^R^)*. Transformed *E. coli* were grown at 37°C with shaking in LB medium (10 g/l tryptone, 5 g/l yeast extract, 10 g/l NaCl, 1 g/l dextrose, and 1 g/l MgCl_2_, pH 7.2) supplemented with ampicillin (100 µg/mL) and chloramphenicol (34 µg/ml) to an OD_600_ of 0.5–0.8. Heterologous protein expression was induced by the addition of 0.3 mM IPTG and the induced cultures incubated at 15°C overnight with shaking. All subsequent purification steps were performed on ice or at 4°C unless otherwise stated.

After overnight induction the cells were harvested by centrifugation and resuspended in 50 mL Chitin Buffer (20 mM Tris-HCl, pH 8 containing 500 mM NaCl, and 0.1 mM EDTA) and lysed by sonication. The supernatants were applied to a 50 mL bed volume chitin resin (New England Biolabs, Inc.) pre-equilibrated with Chitin Buffer. Unbound protein was washed off with at least 10 column bed volumes of Chitin Buffer. The *Mxe* GyrA intein-tag cleavage was induced by a quick column wash with 3 bed volumes of Chitin Buffer supplemented with 30 mM DTT. The intein-tag cleavage reaction was allowed to proceed overnight at 15°C. Fractions from the chitin resin that contained protein were pooled and diluted in Buffer A (composition 20 mM Tris-HCl, pH 8, 0.1 mM EDTA and 5%glycerol). The diluted fractions were applied to a Q column (GE Heathcare Bio-sciences Corp. Piscataway, NJ) and eluted with a 0.05 to 1 M NaCl gradient in buffer 20 mM Tris-HCl, pH 8, 0.1 mM EDTA and 5%glycerol. Fractions containing protein were pooled and dialyzed against a storage buffer of 20 mM Tris-HCl, pH 8 containing 400 mM KCl, and 50% glycerol and stored at −20°C. Protein purity was determined by 10–20% gradient SDS-PAGE gels (Invitrogen™, Carlsbad, CA).

### Endonuclease assays

Endonuclease activity was assayed by incubating various amounts of indicated enzyme in Reaction Buffer (20 mM HEPES-KOH, pH 7.6 containing 23 mM KCl 1 mM DTT, and 2% glycerol) supplemented with MnSO_4_, NaCl or adenine nucleotides where indicated. Unless otherwise stated, ATP and MnSO_4_ were present at 1 mM. For the single time point enzyme titration assays the experiments contained 3.5 nM supercoiled pBR322 and were incubated for 1 hour at 37°C. The reaction was stopped by adding 6X Blue Loading Buffer (0.2% SDS and 50 mM EDTA, pH 8.0, 50% glycerol, 0.02% bromophenol blue) to give a 1X final concentration. The products were resolved on a 1% TEA agarose gel containing 0.1 µg/ml ethidium bromide. DNA species (nicked, supercoiled and linear) were quantified using imageJ® software available from the NIH.

The initial rates were determined at different pBR322 concentrations (1.75 nM to 35 nM) in the Reaction Buffer containing ATP, AMPPNP or ADP as indicated. The reaction was started by adding 2 nmole MutL to the 300 µL reaction to reach the final concentration of 6.6 µM of Aae MutL. Reaction aliquots were taken every 20 seconds and quenched using 6X Blue Loading Buffer. The reaction time points were resolved by agarose gel electrophoresis and visualized by ethidium bromide staining. DNA species (nicked, supercoiled and linear) were quantified using imageJ® software.

### Nucleotide binding assay

Nucleotide binding was measured using a standard filter-binding assay. 100 µL reactions containing 0.98 µM Aae MutL were incubated with varying concentrations of a mixture of cold and ^3^H nucleotide in NEBuffer 2 (10 mM Tris-HCl, pH 7.9, containing 50 mM NaCl, 10 mM MgCl_2_, and 1 mM Dithiothreitol) supplemented with 2.5 µg of single stranded or double stranded M13mp18 phage DNA (New England Biolabs, Inc.) or without DNA, as indicated, for 30 min at room temperature. 20 µl of the reaction was spotted under vacuum onto a Protran® membrane (Whatman Gmbh, Dassel Germany) using a dot-blot manifold apparatus (Whatman) and washed with 500 µl ice cold NEBuffer 2 under vacuum before the membrane was air dried. Each spot was made in triplicate and were counted 3 times for 1 minute on a PerkinElmer Tri-Carb 2900 TR scintillation counter. A standard curve was made using 20 µl of the reaction used in the filter-binding assay.

### MutL C-terminal sequence alignments

The C-terminal amino acid residues of MutL homologues from *Thermotoga maritime*, *Thermoanaerobacter tengcongensis*, *Aquifex aeolicus*, *Thermus aquaticus* and *Homo sapiens* were aligned using ClustalW (http://www.ebi.ac.uk/Tools/clustalw/index.html).

## Results

### ATP modulates Aae MutL endonuclease activity

Previous studies found that hMutLα and yMutLα display non-specific endonuclease activity that is triggered by Mn^++^ and requires the presence of ATP[12], [13]. Furthermore, bacterial homologues of the hPMS2 subunit of hMutLα from *A. aeolicus* and *T. thermophilus* were also reported to posses Mn^++^ induced endonuclease activity [21]. However, ATP was found to act as a repressor of activity in these bacterial homologues. To investigate this phenomenon further, we overexpressed Aae MutL in *E. coli* and purified the enzyme to >95% purity as assessed by SDS-PAGE. Activity of the purified Aae MutL was assayed by following the nicking induced relaxation of supercoiled pBR322.

**Figure 1:**
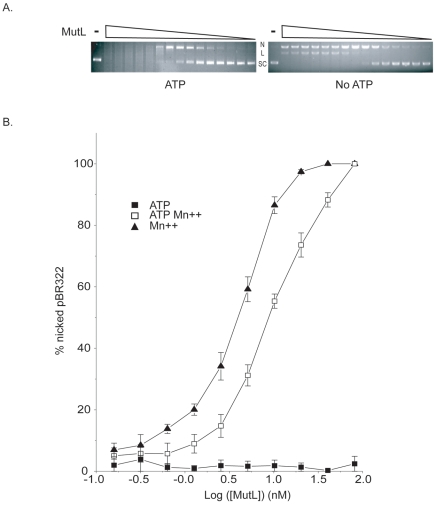
Endonuclease activity titration of Aae MutL. A, Representative data for the nicking of supercoiled pBR322 by various Aae MutL concentrations. The reactions were performed in the presence of 1 mM Mn^++^ and with (left hand panel) or without (right hand panel) 1 mM ATP. The reaction products were resolved on a 1% agarose gel and supercoiled (SC), nicked (N), and linear (L) forms of the plasmid were visible. The control reaction (-) contained supercoiled pBR322 incubated for 1 hour at 37°C in the absence of MutL. The reactions with 2-fold serial dilutions of MutL from 2600 nM (lane 2) to 0.159 nM (lane 16) were incubated for 1 hour at 37°C. B, Graphical representation of agarose gel data showing the relaxation of pBR322 by different amounts of Aae MutL. The enzyme titration was performed as in Figure 1A with 1 mM Mn^++^ (solid triangle), 1 mM ATP (solid square), or 1 mM Mn^++^ and 1 mM ATP (open square). Each point represents the average of 3 separate experiments.

As expected, Aae MutL nicking activity required the presence of Mn^++^ (Figure 1). The nicking assay was performed in the presence or absence of ATP at constant pBR322 concentration for 1 hour at 37°C with Aae MutL serially diluted from 2.6 µM to 0.16 nM (Figure 1). Reaction outcomes were visualized by application to an agarose gel containing ethidium bromide (Figure 1A). Agarose gel band intensities were quantified using ImageJ® software and the data represented graphically (Figure 1B). As expected the conversion of the supercoiled pBR322 to the nicked form occurred at lower Aae MutL concentrations in the absence of ATP as compared to the presence of ATP (Figure 1B). However, at the higher Aae MutL concentrations in the presence of ATP the plasmid was highly degraded when compared to equivalent enzyme concentrations in the absence of ATP. Typically, increased plasmid degradation indicates increased non-specific endonuclease activity.

**Figure 2:**
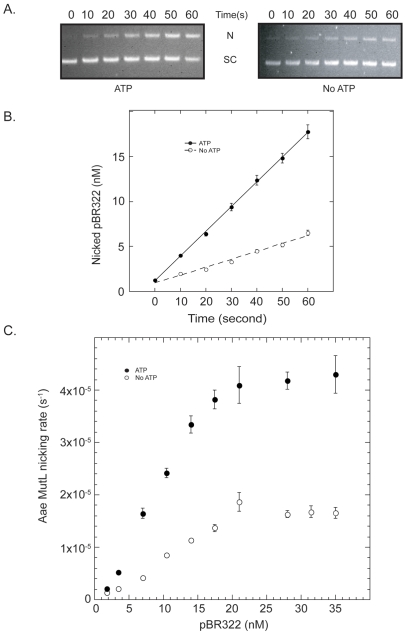
ATP effect on the rate of MutL nicking activity. Reaction time courses of Aae MutL (6.4 µM) with different concentrations of supercoiled pBR322 plasmid. A, Representative data of MutL induced pBR322 (35 nM) nicking time courses with (left hand panel) or without (right hand panel) ATP. An aliquot of the reaction was removed and quenched every 10 seconds as described in the Materials and Methods. B, Bands from the agarose gel time courses were quantified using ImageJ software and the concentration of nicked plasmid was plotted against time. The nicking time courses were generated in the presence (solid circles) and absence (open circles) of ATP. C, The initial nicking rates of Aae MutL were plotted against the concentration of pBR322 used during the time course. The MutL nicking activity was measured with (solid circles) or without (open circles) ATP. Individual points represent the average of 3 separate experiments. Error bars that are not visible are covered by the point marker.

This potential contradiction was investigated by determining initial reaction rates and maximum reaction rate values for the Aae MutL nicking activity. Relaxation of supercoiled pBR322 was followed with time with reaction visualization by agarose gel electrophoresis. Ethidium bromide stained band intensities were quantified using ImageJ® software and the data graphed as illustrated in Figure 2A & B. A linear correlation of pBR322 relaxation with time was observed for times of 1 min or less. From this information initial reaction rate values were determined at various plasmid concentrations (Figure 2C). The reaction rates reached a plateau at values of 1.6±0.08×10^−5^ s^−1^ and 4.2±0.3×10^−5^ s^−1^ in the absence and presence of ATP, respectively, indicating that ATP stimulates Mn^++^ induced Aae MutL activity. The ATP stimulation of Aae MutL activity was also observed for a time course performed at 55°C (data not shown).

### ATP effect on MutL activity at various Mn^++^ concentrations

**Figure 3:**
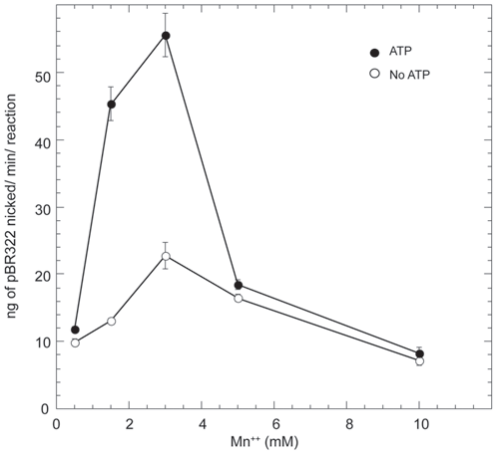
Effect of Mn^++^ concentration on the Aae MutL nicking rate. Initial rate of Mn^++^ induced nicking activity of MutL was measured in the presence (solid circles) or absence (open circles) of ATP at 37°C. Different concentrations of Mn^++^ were added to the buffer solution containing Aae MutL (6.4 µM). The amount of nicked plasmid was quantified with ImageJ software after resolving the different plasmid populations on a 1% agarose gel. Points were taken every 20 seconds for 2 minutes.

The Mn^++^ concentration was varied and the initial rate of the nicking activity on pBR322 was determined in the presence or the absence of 1 mM ATP. The observed nicking rate increased with increasing Mn^++^ concentration up to 3 mM (Figure 3) both in the presence or absence of ATP. Reactions containing 2 and 3 mM Mn^++^ displayed a significant ATP-induced stimulation in endonuclease activity. At concentrations of Mn^++^ above 3 mM the nicking rate decreased both in the presence or absence of ATP, however, the most dramatic drop in activity was seen in the presence of ATP. At the higher Mn^++^ concentrations the rates of nicking were comparable whether ATP was present or not.

### AMPPNP and ADP effect on MutL nicking activity

**Figure 4:**
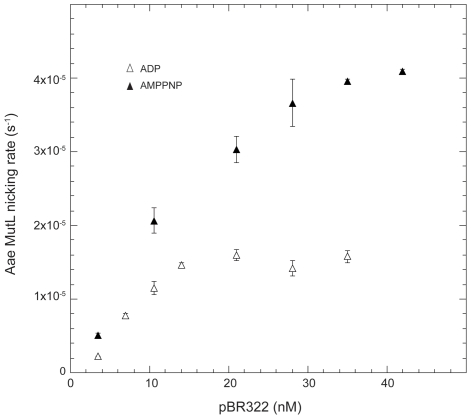
Influence of AMPPNP and ADP on the Aae MutL nicking rate. Initial rates of Mn^++^ induced nicking activity of MutL were measured in the presence of AMPPNP (solid triangles) or ADP (open triangles) at 37°C. Reaction time points were taken every 10 seconds for 2 minutes to determine the initial nicking rate. The initial rates were plotted against the concentration of the plasmid present in the reaction. Individual points are the average of 3 separate reactions.

The importance of ATP hydrolysis in stimulating Aae MutL nicking activity was investigated using the non-hydrolyzable ATP analog AMPPNP. The reactions were performed in the presence of 1 mM AMPPNP. A Michaelis-Menton plot of the Aae MutL nicking rate in the presence of AMPPNP displayed an approximate plateau giving a maximum velocity of 4.1±0.08×10^−5^ s^−1^ (Figure 4). This agreed within experimental error with the maximum velocity determined in the presence of ATP of 4.2±0.3×10^−5^ s^−1^ . The addition of 1 mM ADP did not stimulate MutL activity, giving a maximum velocity of 1.6±0.1×10^−5^ s^−1^ (Figure 4). This is equivalent to the rate of the enzyme with no added nucleotide.

### Aae MutL-CTD is a Mn^++^ induced endonuclease

As demonstrated previously and herein the full-length Aae MutL possesses a Mn^++^ induced endonuclease activity. Furthermore, mutations in conserved regions of Aae MutL-CTD abolish this activity. However, it has not been experimentally demonstrated whether the Aae MutL C-terminal domain itself is sufficient for Mn^++^ induced endonucleolytic activity. To address this question the C-terminal 123 amino acid residues of Aae MutL, residues 303–425, were cloned into a vector for heterologous expression in *E. coli*. To facilitate expression a Met start codon was added to the truncated gene. The enzyme was purified in the same fashion as the full-length Aae MutL to >95% purity as assessed by SDS-PAGE analysis (data not shown).

**Figure 5:**
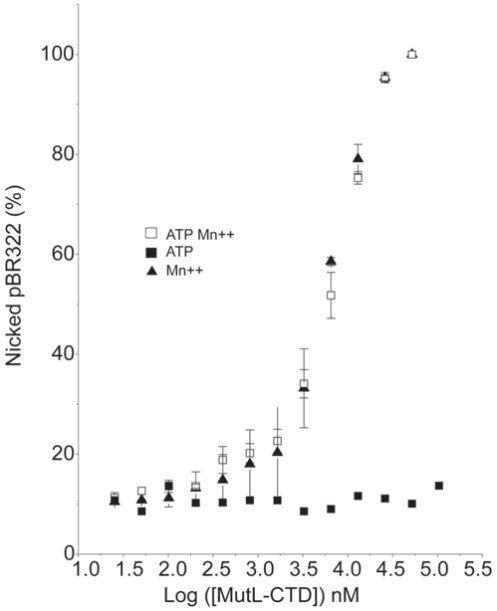
Endonuclease activity of the Aae MutL LC20 fragment. Different concentrations of the Aae MutL C-terminal domain were incubated with 3.5 nM of pBR322 plasmid. The reactions were incubated for an hour at 37°C and stopped by adding loading buffer as described in the Materials and Methods. Reaction products were resolved on a 1% agarose gel. The gel picture was analyzed with ImageJ software. The percentage of nicked plasmid was plotted against the log of the protein concentration. The reaction was performed with 1 mM Mn^++^ (solid triangle), with 1 mM ATP (solid square), or with 1 mM ATP and 1 mM Mn^++^(open square)

Incubation of the Aae MutL-CTD in the absence of Mn^++^ resulted in no detectable relaxation of pBR322 (Figure 5). Addition of 1 mM Mn^++^ to the reaction, however, induced a latent endonuclease activity that was able to completely relax the supercoiled pBR322 at the higher enzyme concentrations. Not surprisingly, the specific activity of the Aae MutL-CTD was apparently lower than the full-length protein because approximately 700 times more Aae MutL-CTD was needed to see plasmid relaxation as compared to the full-length Aae MutL. Unlike the full-length enzyme, Aae MutL-CTD did not display detectable differences in behavior in the presence or absence of ATP (Figure 5). The Mn^++^ induced endonuclease activity of Aae MutL-CTD was observed when the reactions were performed at either 37°C (Figure 5) or 55°C (Figure S2).

### DNA effect on nucleotide binding by full-length and truncated Aae MutL

**Figure 6:**
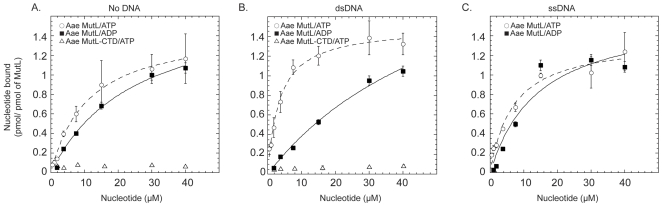
Aae MutL adenine nucleotide binding. The affinity of Aae MutL for different adenine nucleotides was measured by a filter binding assay. The binding of Aae MutL to ATP (open circles) or ADP (solid squares) or of Aae MutL LC20 to ATP (open triangles) was measured. A, nucleotide binding in the presence of dsDNA. B, Influence of ssDNA on full-length Aae MutL nucleotide binding. C, the binding of Aae MutL and Aae MutL LC20 to adenine nucleotides in the absence of added DNA. Each point represents the average of 3 separate experiments.

A filter-binding assay was used to investigate Aae MutL nucleotide binding alone or in the presence of ssDNA or dsDNA (Figure 6). In the absence of DNA or in the presence of ssDNA MutL binds both ATP and ADP with about equal affinity. In the presence of phage M13 dsDNA, however MutL displayed an approximately 2-fold increase in affinity for ATP.

The N-terminal truncation mutant, Aae MutL-CTD, does not possess an identified ATP binding site by sequence alignment. Experimentally the Aae MutL-CTD protein did not bind detectable levels of ATP under the same conditions as used for the full-length enzyme (Figure 6).

### Salt effect on Aae MutL activity

Human MutLα endonuclease activity was inhibited at salt concentrations above 125 mM and this inhibition may be caused by a decrease in its DNA affinity in higher ionic strength solutions[13]. This salt sensitivity may be one reason 23 mM NaCl was used in the human MutLα reaction buffer. Maximal Aae MutL endonuclease activity was observed in the range of 30 to 100 mM NaCl (Figure S1). The nicking rate drops quickly when the conditions reach a salt concentration higher than 100 mM.

### Mutagenesis of the conserved DQHA(X)_2_E(X)_4_E and CPHGRP motifs

**Figure 7:**
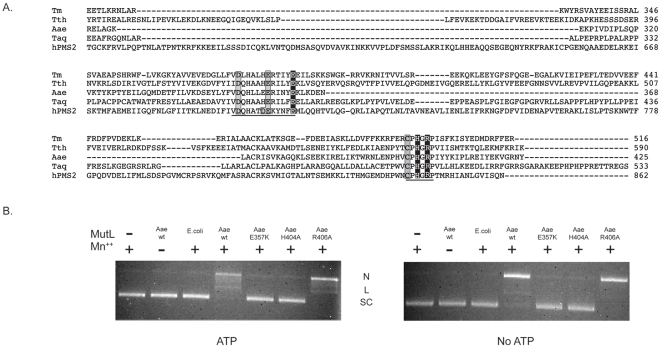
MutL homologue alignment and Aae MutL mutant activity. A, Alignment of the C-terminal amino acid residues of hPMS2 homologues (Tte = *Thermoanaerobacter tengcongensis* (NP_6622976), hPMS2 = human (ABQ59090), Aae = *Aquifex aeolicus* (AAC07483), Taq = *Thermus aquaticus* (EED09553), Tm = *Thermotoga maritima* (AAB09596)). Two regions of high conservation are underlined. The white letters on a black background are the Aae MutL mutants described herein, E for E357K, H for H404A, and R for R406A. The mutations described previously are boxed. B, Endonuclease activities of wild-type and mutant Aae MutL and *E. coli* MutL on supercoiled pBR322 DNA were determined as described in the Materials and Methods. Mn^++^ and ATP were included at 1 mM where indicated. The expected positions of supercoiled (SC), linear (L), and nicked (N) plasmid are indicated.

The DQHA(X)_2_E(X)_4_E motif is conserved throughout known hPMS2 homologues. Mutation of the first Asp or Glu within this motif has been reported to abolish the endonuclease activity of hMutLα, yMutLα, and Tth MutL. Changing the final Glu to Gln within this motif in hPMS2 does not affect activity in an *in vitro* MMR assay [20]. This residue corresponds to E357 in Aae MutL (Figure 7A). A E357K mutation was made by site directed mutagenesis and the mutant protein expressed and purified to >95% purity as assessed by SDS-PAGE analysis (data not shown). In single time point experiments this mutant displayed no detectable Mn^++^ inducible endonuclease activity. The addition of 1 mM ATP did not alter this outcome (Figure 7B).

Another motif, CPHGRP, is also consistently present in hPMS2 homologues and absent in the *E. coli* MutL protein sequence. Previous work has reported that mutation of the first Cys residue of this motif slows the endonuclease activity and abolished the effect of ATP on Tth MutL. Furthermore, mutations of the His and Arg residues of this motif in hPMS2 inhibited an *in vitro* MMR assay [20]. The His and Arg residues in this motif correspond to H404 and R406 in Aae MutL. Single point mutations were created by site directed mutagenesis to create Aae MutL (H404A) and Aae MutL (R406A). These mutant genes were expressed in *E. coli* and purified to >95% homogeneity as assessed by SDS-PAGE analysis (data not shown). The Aae MutL (H404A) protein did not relax supercoiled pBR322 plasmid DNA under conditions that the wild type protein was fully active (Figure 7B). However, the Aae MutL (R406A) mutant was found to possess Mn^++^ induced endonuclease activity as determined by its ability to relax supercoiled DNA (Figure 7B).

## Discussion

The mismatch repair pathway is a well-orchestrated process that performs DNA maintenance to ensure the fidelity of genome replication [25], [26]. The basic elements of MMR are well conserved across life with a divergence in the mechanism of daughter strand recognition. *E. coli* utilizes a methyl-directed mechanism to determine the daughter strand and humans use a nick-directed mechanism. This report focuses on Aae MutL, a key protein in MMR in the hyperthermophic bacteria *A. aeolicus*, and a bacterial MutL that is more similar to human MutL in activity than it is to the better studied MutL protein from *E. coli*. Despite the similarities between Aae and human MutL proteins, there are reported differences in their response to ATP [13], [21].

Recent studies have shown that eukaryotic MutLα carries an endonuclease activity that is stimulated by ATP [12], [13]. Similarly in *E. coli*, ATP activates MutL that in turn stimulates the endonuclease MutH [27]. Therefore, ATP indirectly stimulates endonuclease activity in *E. coli*. Surprisingly, bacterial MutL homologues that possess nicking activity were reported to diverge from this trend and instead be down regulated by ATP [21]. These contradictory findings suggest the possibility that there exists in some bacteria a different regulation of MMR than the homologous pathway in eukaryotes. In order to more fully understand the effect ATP has in bacteria that use nick-directed mismatch repair we undertook further Aae MutL studies.

In single time point enzyme titration experiments that followed the Mn^++^ induced nicking activity of Aae MutL of supercoiled pBR322, ATP apparently down regulated the Aae MutL activity (Figure 1B). This finding was in agreement with previous reports on *T. thermophilus*, *A. aeolicus* MutL, and Neisseria gonorrhoeae MutL (Ngo MutL) [21], [28]. Unexpectedly, at high MutL concentrations in the presence of ATP the substrate is highly degraded while at the same MutL concentration, but in the absence of ATP, the degradation was significantly reduced (Figure 1A). Increased degradation in these experiments often correlates with increased endonuclease activity. These observations led to a change in assay design from single time point experiments to time courses to determine the initial rate of reaction. Under these new conditions a stimulatory effect of 1 mM ATP is clearly evident at 1, 2 and 3 mM Mn^++^ (Figures 2 and 3). The increased reaction rate in the presence of ATP was not detectable at higher Mn^++^ concentrations.

Experimentation with AMPPNP further reinforced that ATP apparently stimulated the initial nicking rate of the Aae MutL protein (Figure 4). The stimulation in nicking activity would explain the extensive degradation visible at the high MutL concentrations in the presence but not the absence of ATP in Figure 1. One possible explanation for these observations would be that Aae MutL nicking activity is stimulated by ATP concomitant with a change in off rate or processivity.

The assay routinely used to study Mn^++^ induced MutL nicking activity involves the conversion of supercoiled plasmid DNA to a relaxed form. This conversion requires only a single nick. Therefore, even though the plasmid contains many potential nick sites only the nick that converts the plasmid from supercoiled to relaxed form is detected. A protein that remains associated with the plasmid after it is relaxed and continues nicking would be determined to be less active than an equally active, but highly distributive, enzyme that nicks a new supercoiled plasmid with each nicking event. This situation may be occurring if ATP converts Aae MutL protein into a more active, but also less distributive enzyme. The significant conformational changes reported when Aae MutL binds ATP may reflect a change to a less distributive form of the enzyme.

The requirement for ATP hydrolysis and the effect of the ATP hydrolysis product, ADP, on the Mn^++^ induced Aae MutL nicking activity was investigated using the non-hydrolyzable ATP analogue AMPPNP and ADP, respectively. AMPPNP stimulated Aae MutL nicking activity to the same extent as observed with ATP (compare Figures 2 and 4). Therefore, ATP hydrolysis does not appear necessary to stimulate Aae MutL nicking activity. ATP hydrolysis is also not required to activate *E. coli* MutL function [29]. The presence or absence of ADP in the reaction had no detectable effect (compare Figures 2 and 4).

From the experimental data described herein ATP appears to stimulate Aae MutL nicking activity. ATP was reported to be necessary for the Mn^++^ induced nicking activity of hMutL and yMutL [12], [13]. Both of these proteins are involved in nick-directed mismatch repair pathways. ATP also stimulates endonuclease activity in *E. coli* and *Haemophilus influenzae* by activating MutL that in turn activates the MutH endonuclease [30], [31]. This may be a further parallel between the more highly studied methyl directed mismatch repair pathway of *E. coli* and nick-directed mismatch repair. Furthermore, ATP binding to a mutant of *E. coli* MutL that lacks ATPase activity has been reported to stimulate MutS association, MutH endonuclease activity, and UvrD loading whereas a mutant that does not bind ATP did not participate in these activities [29], [32], [33], [34], [35]. Therefore, in *E. coli* ATP binding to MutL may stimulate the early steps in methyl-directed MMR repair and its hydrolysis is a transition to later steps.

MutL homologues, including Aae MutL, that are involved in nick-directed mismatch repair seem to merge the *E. coli* MutL and MutH activities. ATP may serve the same basic function in MMR in *A. aeolicus* as it does in *E. coli*. That function being the stimulation of Aae MutL activity, including endonuclease activity, with subsequent hydrolysis of the bound ATP to ADP by the weak MutL ATPase to transition repair from the early to late stages. Tight regulation of MutL function, particularly the endonuclease activity, would minimize undesirable damage to the genome.

Structural studies of *E. coli* and *Thermatoga maritima* MutL, representing MutL enzymes from both methyl-directed and nick-directed MMR pathways, respectively, have shown that an ATPase domain is located in the protein N-terminus[7], [36]. This GHKL ATPase/kinase super family domain is highly conserved and involves four distinct protein motifs[16]. Previous work has demonstrated that mutation of this conserved domain disrupts ATP stimulation of activity in *E. coli* MutL. A truncation mutant Aae MutL was constructed that consisted only of the C-terminal domain and therefore lacked the conserved N-terminal ATPase domain. The fact that this truncated form of Aae MutL does not bind ATP in both the absence or the presence of dsDNA (see Figure 6) reinforces the importance of the N-terminal domain, but not the C-terminal domain, in ATP binding. The truncated Aae MutL-CTD protein retained Mn^++^ induced DNA nicking activity, but was no longer responsive to ATP. This result is in agreement with a recently published article on the C-terminal domain from Ngo MutL [28]. Therefore, the N-terminal domain may bind ATP and induce a change that alters the nicking activity of the enzyme that resides in the C-terminal domain. Furthermore, the observation that Aae MutL-CTD retained Mn^++^ induced DNA nicking activity demonstrated that the C-terminal domain is sufficient for endonuclease activity. The requirement for a higher concentration of Aae MutL-CTD to observe nicking activity may be due to a reduced affinity for DNA caused by removal of the N-terminal region

Amino acid residues have been identified in the C-terminal domain that were important for hPMS2 homologue endonuclease activity. Human MutLα harboring D699K or E705K mutations displayed no detectable endonuclease activity [13]. Comparable mutations in yMutLα and Tth MutL also abolished endonucleolytic activity [12], [21]. The mutations alter the DQHA(*X*)_2_E(*X*)_4_E motif found in the C-terminal domain of these enzymes and it may be part of a divalent metal cation binding site [13], [20]. A mutation within the final Glu in the DQHA(*X*)_2_E(*X*)_4_E sequence of hPMS2 was reported to have no effect in an *in vitro* MMR assay. Interestingly, the comparable mutation in Aae MutL, E357K, results in a nucleolytically inactive protein (Figure 7).

The CPHGRP motif is another highly conserved region found in hPMS2 homologues. Previous work reported that mutation of the Cys within this motif did not abolish endonuclease activity, although this mutant no longer responded to ATP [21]. Mutation of the conserved His or Arg residue in hPMS2 resulted in impaired *in vitro* MMR [20]. We further investigated the role of this region by creating H404A and R406A mutations in the CPHGRP motif of Aae MutL. Aae MutL (H404A) did not have detectable endonuclease activity, while R406A remained active (Figure 7). Interestingly, the conserved His residue in this motif was previsouly implicated in metal ion binding, but the Arg was not [20]. This region of the protein may be involved in multiple interactions and we concur with the previous statement that a complete structure of a hPMS2 homologue in complex with substrate would facilitate further structure/function studies [21].

In conclusion, our findings suggest that ATP stimulates the endonucleolytic activity of Aae MutL in common with the eukaryotic homologues hMutLα and yMutLα. It is possible that ATP alters other protein properties, such as processivity, that complicates experimental investigation. Furthermore, 2 mutations were identified that abolish Aae MutL endonucleolytic activity. The H404A mutation in the conserved CPHGRP motif abolished Mn^++^ induced endonuclease activity, while R406A did not. Finally, the ATP stimulation of hPMS2 homologues appears to be conserved from at least a subset of bacteria to multicellular eukaryotes.

## Supporting Information

Figure S1 Effect of salt concentration on the initial nicking rate of Aae MuL. Time courses of the Aae MuL nicking activity on pBR322 were performed at NaCl concentrations from 5 mM to 200 mM. The reaction was stopped by the addition of blue loading dye and resolved on a 1% agarose gel. The initial rates were plotted against the corresponding salt concentration.
(9.61 MB TIF)

Figure S2 Titration of the nicking activity of MutL-CTD at 55°C. Representative data for the nicking of supercoiled pBR322 by various Aae MutL-CTD concentrations. The reactions were performed in the presence of 1 mM Mn^++^. The reaction products were resolved on a 1% agarose gel and supercoiled (SC), nicked (N), and linear (L) forms of the plasmid were visible. The control reaction (-) contained supercoiled pBR322 incubated for 1 hour at 55°C in the absence of MutL-CTD. The reactions with 2-fold serial dilutions of MutL-CTD from 33 µM (lane 2) to 257 nM (lane 10) were incubated for 1 hour at 55°C.
(9.38 MB TIF)
