# Supplementary figures and images for: Adenosine Triphosphate Stimulates Aquifex aeolicus MutL Endonuclease Activity
Source: PLoS One. 2009 Sep 24;4(9):e7175. doi: 10.1371/journal.pone.0007175 (PMC2744016; doi:10.1371/journal.pone.0007175)

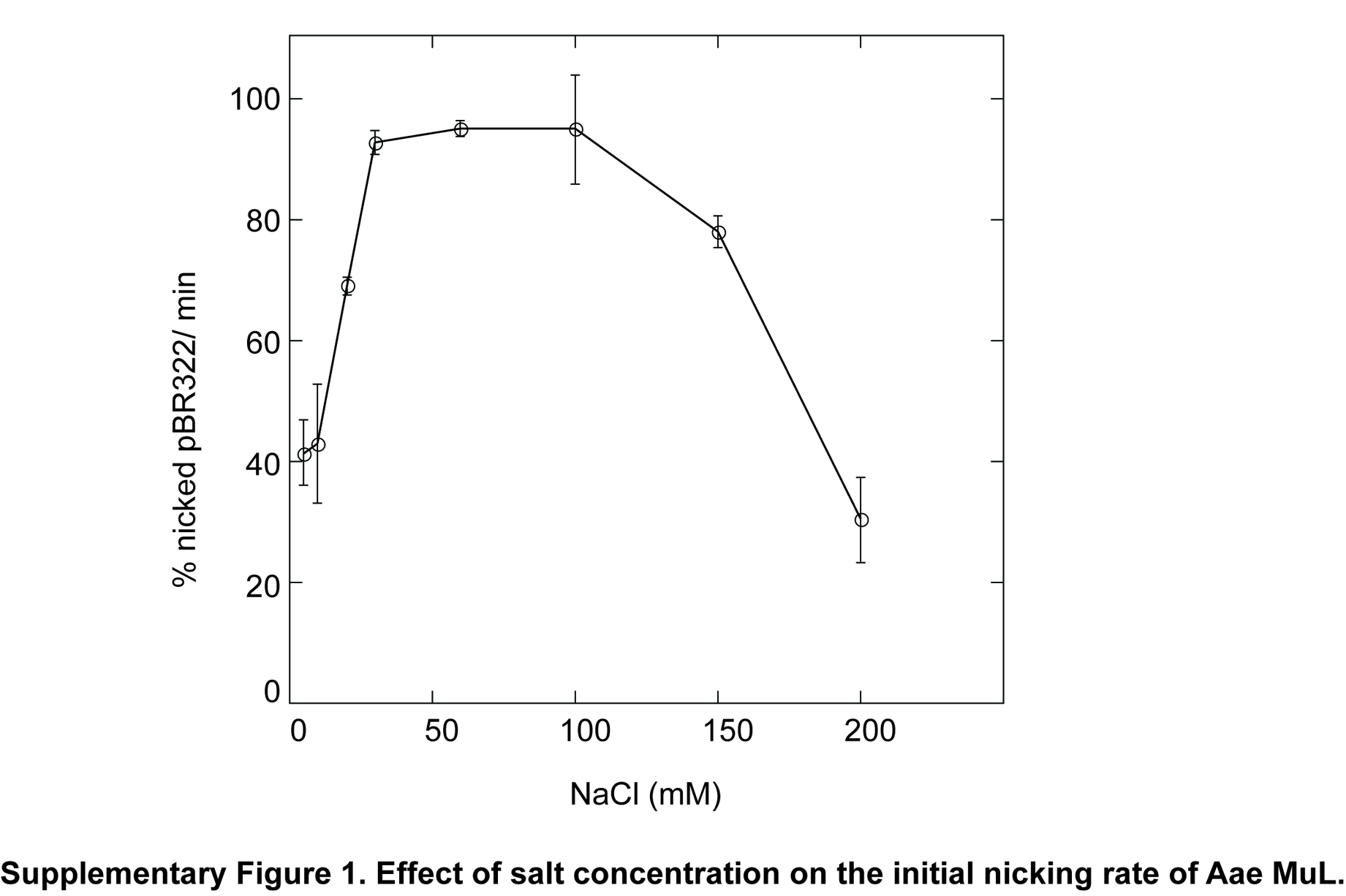

Supplement: Figure S1 — Effect of salt concentration on the initial nicking rate of Aae MuL. Time courses of the Aae MuL nicking activity on pBR322 were performed at NaCl concentrations from 5 mM to 200 mM. The reaction was stopped by the addition of blue loading dye and resolved on a 1% agarose gel. The initial rates were plotted against the corresponding salt concentration. (9.61 MB TIF) [file pone.0007175.s001.tif]

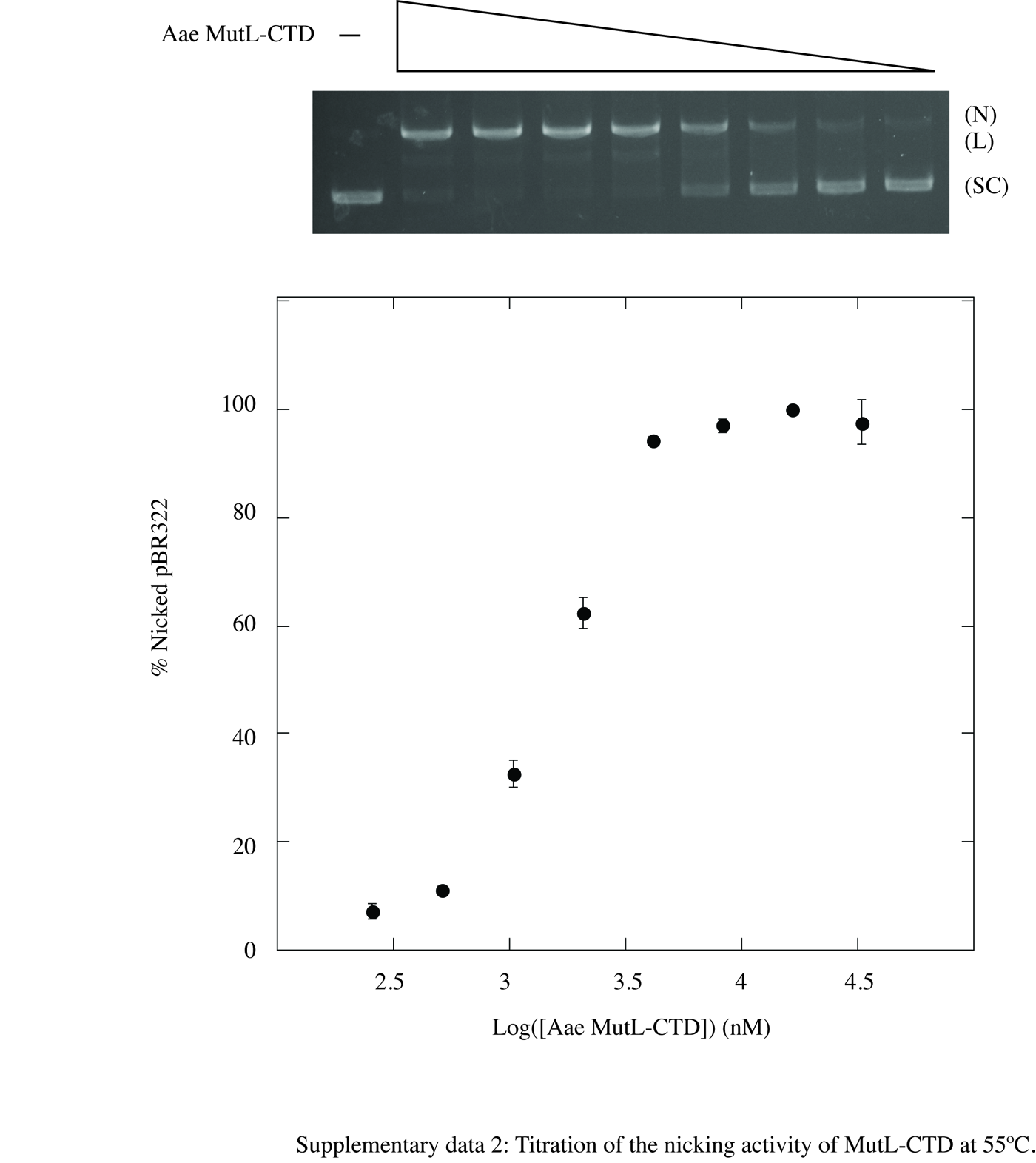

Supplement: Figure S2 — Titration of the nicking activity of MutL-CTD at 55°C. Representative data for the nicking of supercoiled pBR322 by various Aae MutL-CTD concentrations. The reactions were performed in the presence of 1 mM Mn++. The reaction products were resolved on a 1% agarose gel and supercoiled (SC), nicked (N), and linear (L) forms of the plasmid were visible. The control reaction (-) contained supercoiled pBR322 incubated for 1 hour at 55°C in the absence of MutL-CTD. The reactions with 2-fold serial dilutions of MutL-CTD from 33 µM (lane 2) to 257 nM (lane 10) were incubated for 1 hour at 55°C. (9.38 MB TIF) [file pone.0007175.s002.tif]
